# Relationship Between Fragmented QRS Complex and Left Ventricular Fibrosis and Function in Patients With Danon Disease

**DOI:** 10.3389/fcvm.2022.790917

**Published:** 2022-02-21

**Authors:** Jiajun Xie, Yang Liu, Xiaoyu Wei, Weitao Ye, Zelan Ma, Guanyu Lu, Zekun Tan, Tingyu Li, Yining Wang, Lei Zhao, Minjie Lu, Xiaohu Li, Yucheng Chen, Hui Liu

**Affiliations:** ^1^The Second School of Clinical Medicine, Southern Medical University, Guangzhou, China; ^2^Department of Radiology, Guangdong Provincial People's Hospital, Guangdong Academy of Medical Sciences, Guangzhou, China; ^3^Guangdong Cardiovascular Institute, Guangdong Provincial People's Hospital, Guangdong Academy of Medical Sciences, Guangzhou, China; ^4^Department of Radiology, Sun Yat-sen Memorial Hospital, Sun Yat-sen University, Guangzhou, China; ^5^Department of Radiology, The Second Affiliated Hospital of Guangzhou University of Chinese Medicine, Guangzhou, China; ^6^Department of Radiology, State Key Laboratory of Complex Severe and Rare Diseases, Peking Union Medical College Hospital, Chinese Academy of Medical Science and Peking Union Medical College, Beijing, China; ^7^Department of Radiology, Beijing Anzhen Hospital, Capital Medical University, Beijing, China; ^8^Department of Magnetic Resonance Imaging, Fuwai Hospital and National Center for Cardiovascular Diseases, Chinese Academy of Medical Sciences and Peking Union Medical College, Beijing, China; ^9^Department of Radiology, The First Affiliated Hospital of Anhui Medical University, Hefei, China; ^10^Department of Cardiology, West China Hospital, Sichuan University, Chengdu, China; ^11^Guangdong Provincial Key Laboratory of Artificial Intelligence in Medical Image Analysis and Application, Guangdong Provincial People's Hospital, Guangdong Academy of Medical Sciences, Guangzhou, China

**Keywords:** Danon disease, myocardial fibrosis, electrocardiography, fragmented QRS, cardiac magnetic resonance imaging (CMR)

## Abstract

**Background:**

Fragmented QRS (f-QRS) complex on the surface ECG is a cardiac conduction abnormality that indicates myocardial scarring. The relationship between the f-QRS complex and cardiac status in patients with Danon disease (DD) remains unclear and will be explored in this study.

**Methods:**

Patients with genetically confirmed DD and cardiac magnetic resonance imaging (CMR) examinations were recruited from multiple centers. The number of leads, pattern, score, and segmental distribution of the f-QRS complex were assessed by surface 12-lead ECG. Cardiac status, such as left ventricular (LV) volume, function, and extent of late gadolinium enhancement (LGE), was demonstrated by CMR. The segmental distribution of LGE was also assessed. Correlations between the f-QRS and cardiac status were assessed.

**Results:**

Fifteen patients (14 men) with DD who underwent 12-lead ECG and CMR imaging were included. The f-QRS complex was documented in all patients (*n* = 15, 100%). Three patterns of f-QRS were found, with the notched R/S pattern (74%) being the most common, followed by fragmented QRS (16%) and various RSR' (11%). The fragmented QRS pattern showed an association with a higher level of myocardial fibrosis (LGE > 35%). The burden of f-QRS in each patient was assessed by the number of leads with f-QRS (median 7, range 2–12) and the f-QRS score (median 9, range 2–33). In the correlation analysis, the f-QRS score was positively correlated with LGE% (*r* = 0.726, *p* = 0.002), negatively correlated with LV ejection function (LVEF; *r* = −0.617, *p* = 0.014) as evaluated by CMR. In the local distribution, f-QRS score and LGE% were both predominant in the LV free wall but did not correlate well among the anterior, lateral, and inferior segments.

**Conclusion:**

In this DD cohort, the quantitative f-QRS was correlated well with myocardial fibrosis burden and LV dysfunction in general. This finding suggests that f-QRS can be used as a simple screening tool to assess cardiac status in patients with DD.

## Introduction

Danon disease (DD) is a rare X-chromosome-linked genetic disorder caused by a lysosome-associated membrane protein-2 (LAMP2) gene mutation (1). Due to the accumulation of intracytoplasmic autophagic vacuoles in the myocardium, many patients with DD cardiomyopathy usually develop in their childhood or young adulthood severe conditions, such as fatal cardiac hypertrophy, progressive heart failure, and often ventricular arrhythmias (2, 3). The identification and monitoring of Danon cardiomyopathy are important for the timing of clinical interventions that may include defibrillator placement and heart transplantation.

Cardiac magnetic resonance imaging (CMR) is an excellent imaging technique for morphological recognition, functional evaluation, and tissue characterization (4, 5). Late gadolinium enhancement (LGE) at CMR usually reflects irreversible myocardial fibrosis or scarring (6, 7). The typical pattern of LGE in DD cardiomyopathy is generally characterized by an extensive distribution in the left ventricular (LV) free wall and usually sparing or relatively less involved in the interventricular septum (8–11).

The 12-lead ECG is the most frequently utilized and validated non-invasive diagnostic test to assess electrocardiographic abnormalities. The fragmented QRS (f-QRS) complex, generally defined as notching in the R wave or the S wave (notched R/S) and double or multiple R spikes, which have been widely reported as an ECG marker of myocardial fibrosis in various heart diseases and has a prognostic value (12–17). However, thus far, there have been no data regarding the relationship between f-QRS complex and cardiac status assessed by CMR in a DD cohort.

Therefore, the purpose of this study was to evaluate the relationship between the f-QRS complex and LV electroanatomic substrate abnormalities assessed by CMR in patients with DD.

## Methods

### Patient Population

The study protocol was approved by the local institutional review board and written informed consent was waived. Fifteen patients with genetically confirmed DD were recruited retrospectively, from January 2010 to May 2020, from six tertiary centers in China. All patients underwent CMR scanning and 12-lead ECG examinations. ECG was recorded before CMR with a 0.1 to 1.0-month time interval. Of the 15 patients, 14 had previously reported CMR results to our colleagues (9). Demographic and clinical data were also collected, such as sex, age, symptoms, duration from symptom onset, genetic results, and the latest follow-up information.

### Electrocardiography and f-QRS Complex

**Figure 1:**
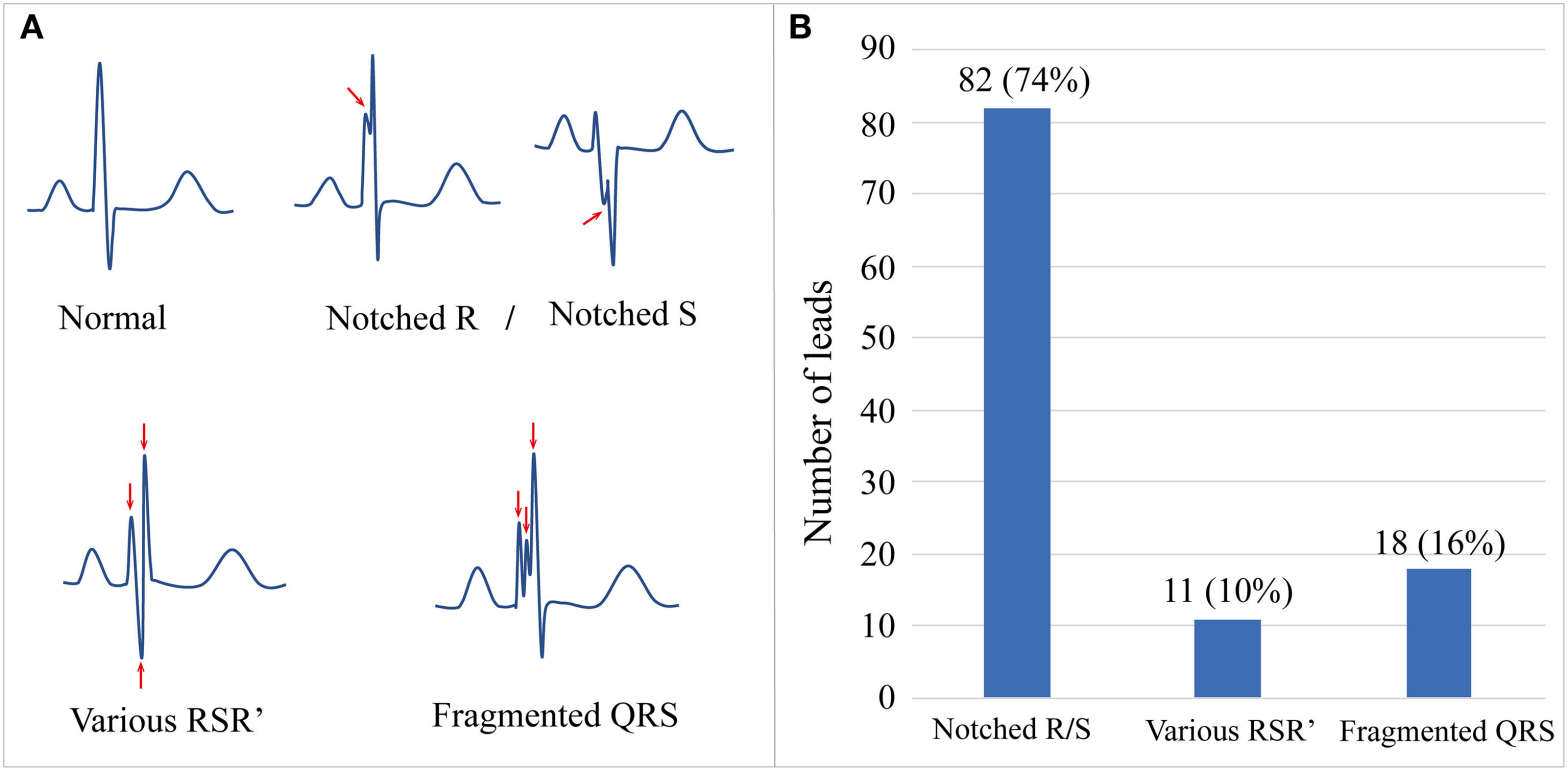
The schematic diagram and prevalence of f-QRS complex in our Danon disease cohort. **(A)** Schematic diagrams show the normal morphology of QRS and different patterns of f-QRS complexes. **(B)** The number and proportion of three main patterns of f-QRS in our cohort. f-QRS, fragmented QRS.

The results of a standard 12-lead ECG (paper speed, 25 mm/sec, 10 mm/mV) were collected and analyzed by an experienced cardiologist. Using the criteria proposed by Das et al. (17), f-QRS complexes were defined as QRS waves with notching in the R wave or S wave (notched R/S) or an additional R' wave, such as RSR', rSr', rSR', (various RSR'), or >2 R waves or 2 notches (fragmented QRS) in two contiguous septal leads (V1–V2), anterior leads (V3–V4), lateral leads (I, aVL, V5, and V6), or inferior leads (II, III, and aVF) (Figure 1). The wide f-QRS complex was defined to f-QRS with QRS duration >120 ms. The personal and total number of leads with different f-QRS complexes were counted. The f-QRS score method was used to evaluate f-QRS burden for each patient by adding the score of the first QRS in the 12 leads (notched R/S: 1 score; various RSR': 2 scores; f-QRS: 3 scores). The segmental f-QRS score was calculated by averaging the f-QRS score of the septal (V1–V2), anterior (V3–V4), lateral (I, aVL, V5, and V6), and inferior leads (II, III, and aVF). Ventricular pre-excitation was defined as the presence of a short PR interval (<120 ms) with a widened QRS complex (>110 ms) or an abnormal initial QRS vector (delta wave).

### CMR Protocol

#### MR Protocol

All CMR images were obtained with a 3.0 T and/or 1.5 T scanner using standard protocols. Breath-hold cine-balanced steady-state-free precession functional images were acquired in short-axis and two-, three- and four-chamber views. Black blood images were obtained using breath-hold T2-weighted and T2-short-tau inversion recovery (STIR) images in cardiac short-axis orientations. Perfusion images were acquired using a saturation-recovery sequence in vertical and horizontal long- and short-axis orientations. LGE was performed 10 min after administration of an intravenous bolus of gadolinium, using a phase-sensitive inversion recovery sequence. The LGE imaging inversion time was individually adapted to null the signal of the remote myocardium. The detailed CMR acquisition protocol and sequences from different centers are shown in Supplementary Table 1.

### Imaging Analysis

All CMR images were analyzed in our center through consensus by two experienced radiologists blinded to the clinical data, using commercially available software (Medis 3.0; Medis Imaging Systems). Endocardial and epicardial borders were outlined manually on all end-diastolic and end-systolic short-axis cine slices. LV end-systolic and end-diastolic volumes, LV ejection fraction (EF), and LV mass were then calculated in a standard fashion. LV maximal wall thickness was automatically measured using the software. The hypertrophic cardiomyopathy (HCM) and dilated cardiomyopathy (DCM) phenotypes were evaluated according to the guidelines (18, 19). LGE was defined as 5 SD above the mean signal intensity of an apparently normal myocardium. The extent of LGE was assessed according to the sum of enhanced areas on all short-axis images (LGE mass; expressed as g) and the proportion of LV mass (%LGE, expressed as %). The main location of the LGE was recorded as the LV free wall and interventricular septum. The pattern of LGE was defined as extensive (involving ≥3 segments continuously) and/or patchy (involving <3 segments discontinuously). Another four-segment method, such as the septal (S2, S3, S8, S9, and S14), anterior (S1, S7, and S13), lateral (S5, S6, S11, S12, and S16), and inferior (S4, S10, and S15) segments, was used to assess the mean LGE% in the corresponding four-lead segments on ECG.

### Statistical Analysis

The data were analyzed using statistical software (SPSS, version 27.0; IBM). Continuous variables are expressed as medians and ranges, and categorical variables are expressed as frequencies and proportions. Because the number of leads of f-QRS and f-QRS scores does not follow a normal distribution, the correlations between f-QRS and CMR parameters were calculated using Spearman's rank correlation. Fisher's exact test was used to compare the percentages between the subgroups. Differences were considered significant if the *p* was < 0.05.

## Results

### Demographic Data

Fifteen patients with DD from six tertiary hospitals were included (14 men [93%]; median age, 19 years; age range, 14–44 years). The clinical information, ECG, and CMR parameters of the patients are listed in Table 1. The genetic mutation information and additional details are shown in Supplementary Table 2.

**Table 1:** Baseline characteristics.

	***N* = 15**
**Demographics**	
Age (years)^*^	19 (14, 44)
Sex (male)	14 (93)
Symptoms	
Chest pain	5 (33)
Shortness of breath	8 (53)
Syncope	6 (47)
Duration from symptom onset (years)^*^	1 (1, 9)
Follow-up	
ICD	1 (7)
RFA	3 (20)
Death	6 (40)
**ECG**	
f-QRS counts
Base on patients	15 (100)
Base on leads^*^	7 (2, 12)
f-QRS score^*^	9 (2, 33)
PR interval (ms)^*^	97 (80, 162)
QRS duration (ms)^*^	150 (100, 240)
QTc interval (ms)^*^	470 (341, 615)
Atrial fibrillation	3 (20)
Preexcitation	13 (87)
Conduction block	3 (20)
**Imaging**	
LV EF (%)^*^	32 (8, 74)
LV EDVi (ml/m^2^)^*^	149 (92, 321)
LV ESVi (ml/m^2^)^*^	102 (32, 261)
LV MWT (mm)^*^	21 (11, 33)
LV Mi (g/m^2^)^*^	256 (88, 477)
HCM phenotype	14 (93)
DCM phenotype	1 (7)
LV LGE mass (g)^*^	96 (8.5, 390)
LV LGE (%)^*^	35 (4, 60)
LGE location	
LV free wall	15 (100)
LV septum	10 (67)

* *Data are medians, and data in parentheses are ranges. Unless otherwise indicated, data are numbers of patients, and data in parentheses are percentages. f-QRS, fragmented QRS; ICD, implantable cardioverter defibrillator; RFA, radiofrequency ablation; LV, left ventricle; EF, ejection fraction; EDVi, end-diastolic volume index; MWT, maximum wall thickness; Mi, mass index; HCM, hypertrophic cardiomyopathy; DCM, dilated cardiomyopathy; LGE, late gadolinium enhancement*.

### f-QRS on 12-Lead ECG

Fragmented QRS complexes were documented in all patients (*n* = 15, 100%). The total number of leads with f-QRS was 111. The median number of leads with f-QRS was 7 (range 2–12), and the median f-QRS score was 9 (range 2–33; Table 1). Figure 1 shows three representative patterns of f-QRS, with notched R/S being the most common (82/111, 74%), followed by fragmented QRS (18/111, 16%) and various RSR' (11/111, 11%). Based on patients, the notched R/S was found in all patients (15/15, 100%), the various RSR' was found in 6 (6/15, 40%), and the fragmented QRS were found in five patients (5/15, 33.3%; Table 2). The duration of fragmented QRS was wide in all (*n* = 18), ranging from 180 to 220 ms (median 205 ms).

**Table 2:** The incidence of three kinds of f-QRS complexes in all subjects and different degrees of myocardial fibrosis subgroups.

**f-QRS pattern**	**All patients**	**LGE% <35%**	**LGE%>35%**	*P* value^*^
	**(*n* = 15)**	**(*n* = 7)**	**(*n* = 8)**	
Notched R/S	15 (100)	7 (46.7)	8 (53.3)	-
Various RSR'	6 (100)	1 (16.7)	5 (83.3)	0.119
Fragmented QRS^#^	5 (100)	0 (0)	5 (100)	0.026

* *p according to the exact-Fisher test*.

# *All fragmented QRS complexes were wide f-QRS with a QRS time > 120 ms in this study. Data are numbers of patients, and data in parentheses are percentages*.

*f-QRS, fragmented QRS; LGE, late gadolinium enhancement*.

### Cardiac Status Measured at CMR

In the morphological analysis, 14 patients (13 men and one woman) presented with the HCM phenotype with LV dilatation, and only one male presented with the DCM phenotype. The median of LV maximum wall thickness was 21 mm (range, 11–33 mm). LV ejection function (LVEF) was decompensated in 13 patients (13/15, 86%) with a median value of 32% (range, 8–74%). LGE was detected in all 15 patients with a median LV LGE% of 35% (range 4–60%), which was predominantly involved in the LV free wall (*n* = 15, 100%) and less in the septum (*n* = 10, 67%; Table 1).

### Correlation Between f-QRS and CMR Parameters

**Figure 2:**
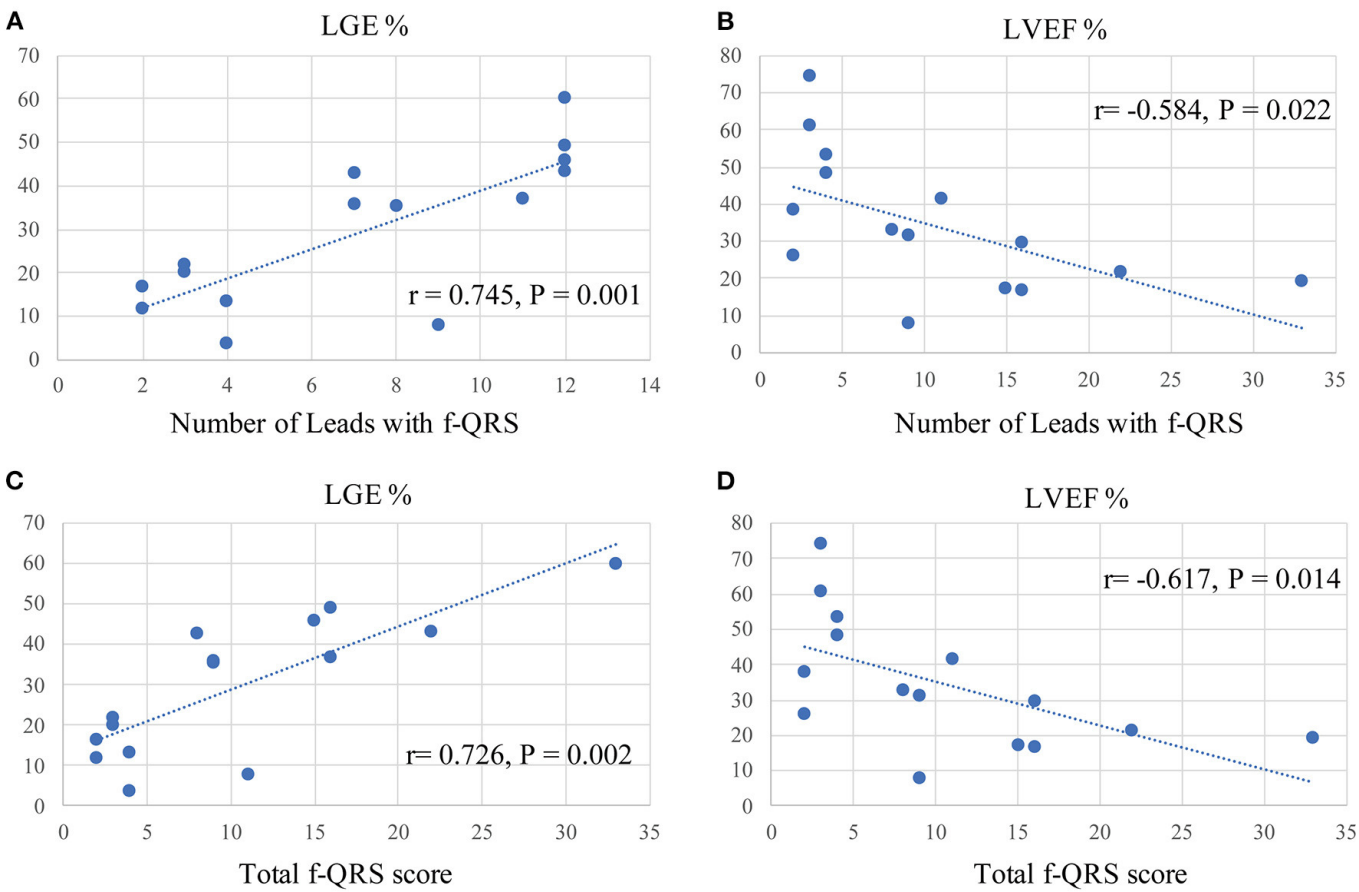
Spearman correlation analysis between f-QRS and CMR parameters in our Danon disease cohort. f-QRS, fragmented QRS; LGE, late gadolinium enhancement. **(A)** Correlation between the number of leads with f-QRS and LGE% (*r* = 0.745, *p* = 0.001). **(B)** Correlation between the number of leads with f-QRS and LVEF% (*r* = −0.584, *p* = 0.022). **(C)** Correlation between the number of leads with f-QRS and LGE% (*r* = 0.726, *p* = 0.002). **(D)** Correlation between f-QRS score with f-QRS and LVEF% (*r* = −0.617, *p* = 0.014).

Spearman correlation analyses were conducted to determine any correlation between the f-QRS and CMR parameters. Figure 2 shows that the number of leads with f-QRS is significantly correlated with LGE% (*r* = 0.745, *p* = 0.001) and negatively correlated with LVEF% (*r* = −0.584, *p* = 0.022). The f-QRS score was also positively correlated with the LV LGE% (*r* = 0.726, *p* = 0.002) and negatively correlated with LVEF% (*r* = −0.617, *p* = 0.014). When dividing the 15 patients into two subgroups by LGE% = 35%, notched R/S was equal in both groups, the various RSR' was almost present in those with LGE% >35% (5/6, 83.3%), and the f-QRS type was only seen on those with LGE% >35% (5/5, 100%), and the variance was statistically significant (*p* = 0.026).

### Segmental Distribution of f-QRS and LGE in DD

**Figure 3:**
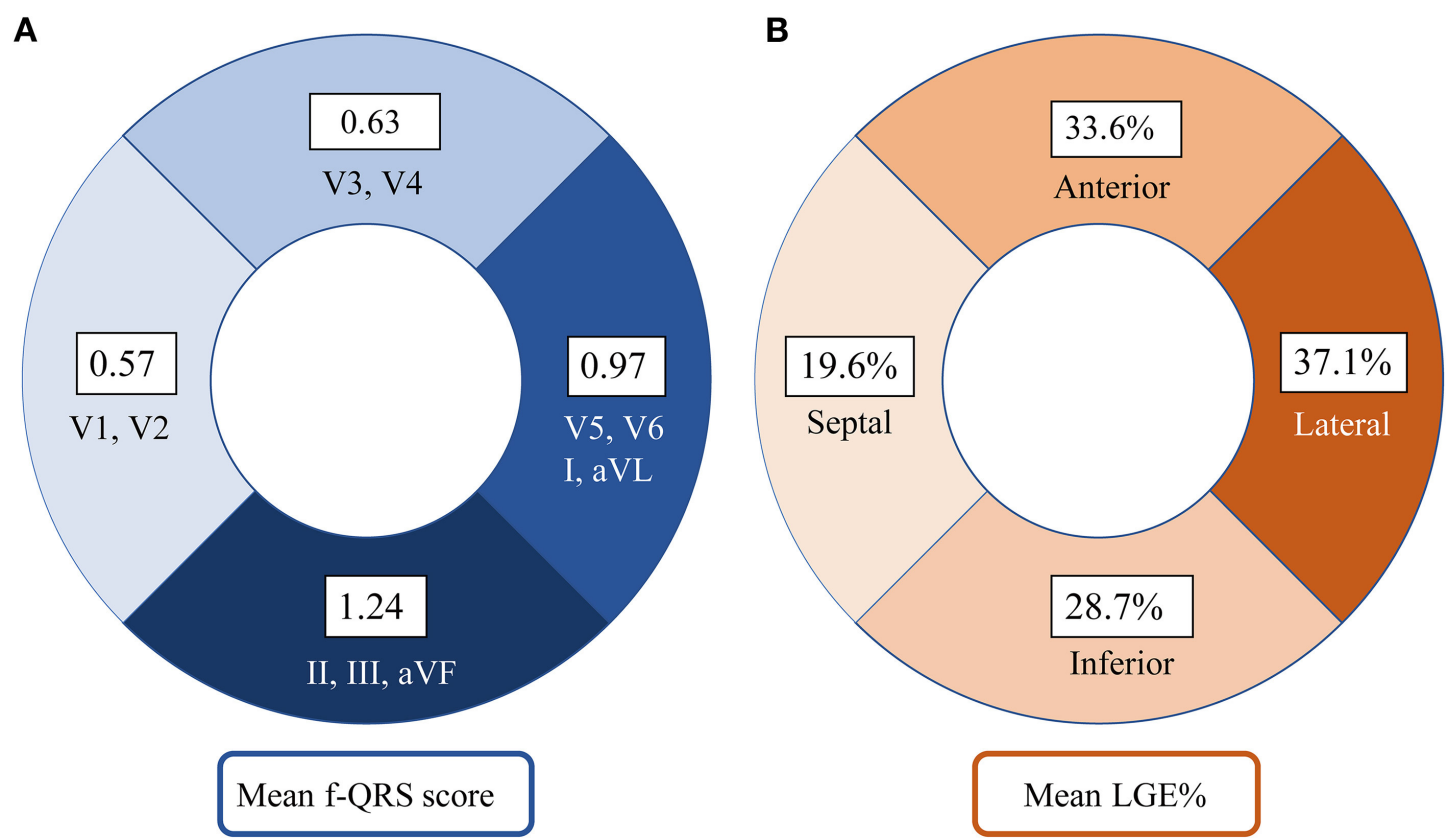
Distribution of f-QRS score and LGE in the left ventricle in our Danon disease cohort. **(A)** The number on the fan-shaped block represents the mean f-QRS score of septal leads (V1–V2), anterior leads (V3–V4), lateral leads (I, aVL, V5, and V6), and inferior leads (II, III, and aVF). **(B)** The number on the fan-shaped block represents the mean LGE% of septal (S2, S3, S8, S9, and S14), anterior (S1, S7, and S13), lateral (S5, S6, S11, S12, and S16), and inferior (S4, S10, and S15) segments. LGE, late gadolinium enhancement; f-QRS, fragmented QRS.

**Figure 4:**
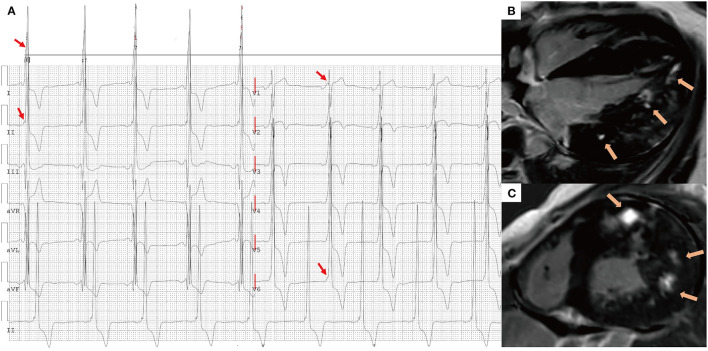
A 16-year-old male presented with chest tightness and shortness of breath and was genetically diagnosed with Danon disease (mutation site: c.936G > A). **(A)** f-QRS with a notched R pattern (red arrows) were found in the lateral leads (I, aVL, V6); the total f-QRS score was 3. **(B)** Four-chamber view and **(C)** short-axis view of LGE images on CMR showed patchy fibrosis/scarring in the lateral wall of LV (orange arrows). Global LV LGE% was 20%. f-QRS, fragmented QRS; LGE, late gadolinium enhancement.

**Figure 5:**
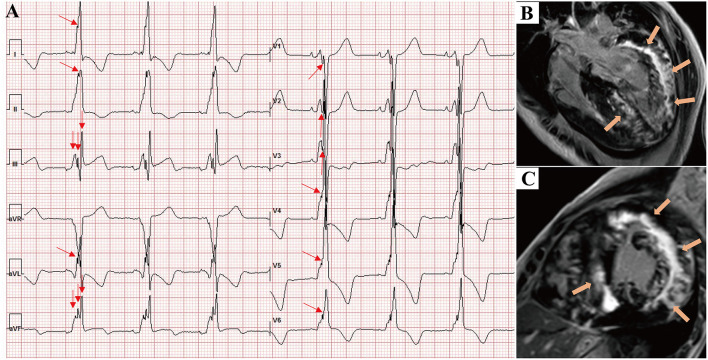
A 17-year-old male presented with chest tightness, shortness of breath, and syncope and was genetically diagnosed with Danon disease (mutation site: c.35C > A). **(A)** f-QRS was found in 12 leads. Notched R/S patterns (single red arrows) were found in the I, II, aVL, and V1–V6 leads. The fragmented QRS pattern was found in III and aVF leads (double red arrows), and the total f-QRS score was 15. **(B)** Four-chamber view and **(C)** short-axis view of LGE images on CMR showed the extensive fibrosis/scarring in the lateral wall of LV and patchy fibrosis in the septum (orange arrows). Global LV LGE% was 36.7%. LGE, late gadolinium enhancement.

Figure 3 shows that the septal wall had the lowest mean f-QRS score (0.57) and extent of LGE (19.6%). In the LV free wall, the mean f-QRS score was the highest in the inferior leads (1.24), followed by the lateral (0.97) and anterior (0.63) leads (Figure 3A). The mean of LGE% was the highest in the lateral segments (37.1%), followed by the anterior (33.6%) and inferior segments (28.7%; Figure 3B). Typical cases that represent the relationship between f-QRS complexes with ECG and LGE at CMR in DD are shown in Figures 4, 5.

### f-QRS Complex Characteristics of DD Patients in the Literature

Supplementary Table 3 summarizes previously reported clinical data and 12-lead ECG characteristics of patients with DD. A total of 27 studies with 36 patients (27 men and nine women) were reported between 2005 and 2020. The f-QRS complexes were observed in 31 (31/36, 86%) patients with 12-lead ECGs, with a total number of 168. The median f-QRS score in these 36 patients was 7 (range 0–17). The pattern and segmental distribution of f-QRS were similar to those in our cohort. Notched R/S was the most common pattern (146/168, 87%), followed by fragmented QRS (12/168, 7%) and various RSR' (10/168, 6%; Supplementary Figure 1A). In the segmental analysis, the septal leads also had the lowest mean f-QRS score (0.47); in the LV free wall, the mean of f-QRS score was the highest in the inferior leads (0.74), followed by the lateral (0.58) and anterior (0.53) leads (Supplementary Figure 1B). Of the 36 cases, six (11, 13, 16, 19, 22, and 35 in Supplementary Table 3) had LGE on CMR examination, together with f-QRS complexes presented on their ECGs.

### Relationship Between f-QRS Complex and Outcome of Patients With DD

In our cohort (*n* = 15), the mortality incidence was lower in patients with an f-QRS score <9 (2/7, 29%) than in those with f-QRS score ≥9 (4/8, 50%), although the variance was not statistically significant (Supplementary Table 4).

In the literature cohort (*n* = 36), the mortality incidence was lower in patients with an f-QRS score <9 (4/23, 17%) than in those with an f-QRS score ≥9 (6/13, 46%); invasive treatments were recorded less in patients with an f-QRS score <9 (6/23, 26%) than in those with an f-QRS score ≥9 (7/13, 54%). However, the difference was not statistically significant (Supplementary Table 4).

## Discussion

In this study, we found that the f-QRS and its variants were commonly seen in patients with DD, both in our group (100%) and the literature cohort (86%). The fragmented QRS pattern showed an association with a higher level of myocardial fibrosis (LGE >35%). Furthermore, the quantitative f-QRS score showed a positive relationship with myocardial fibrosis (LGE%) and a negative relationship with LV systolic function (LVEF%). Unexpectedly, the local distribution of f-QRS score and LGE% was predominant in the LV free wall but did not correlate well among the segments (anterior, lateral, and inferior). To our knowledge, this is the first study to evaluate the relationship between f-QRS on surface ECG and cardiac status on CMR imaging in patients with DD.

### Relationships Between f-QRS and the Extent of Myocardial Fibrosis

A close relationship between f-QRS and myocardial fibrosis was observed in our patients with DD. This finding was consistent with several studies on other cardiac conditions (14, 17, 20, 21), which demonstrated that f-QRS was useful in estimating myocardial fibrosis as assessed by CMR LGE. However, other cardiac conditions showed a generally lower occurrence rate of f-QRS on ECG, such as myocarditis (23–76%) (16, 22), HCM (56–63.89%) (20, 23), and LV non-compaction cardiomyopathy (48%) (24). The positive relationship between f-QRS and LGE suggests that the fibrosis/scarring involved the myocardium in our patients with DD more severely than the cardiac condition mentioned above. Among the three main patterns of f-QRS in our cohort, the fragmented QRS type, as a wide f-QRS defined by Das et al. (17), seems a more malignant marker associated with severe myocardial fibrosis (LGE > 35%), similar to the finding from Das et al. (12), who found that wide f-QRS was a moderately sensitive and highly specific sign for myocardial scar in patients with known or suspected coronary artery disease.

The underlying mechanism of myocardial fibrosis progression in DD remains unclear. It has been demonstrated that the reduction in LAMP2 protein disrupts intracytoplasmic trafficking, which leads to the accumulation of autophagic materials in myocardial cells (1, 25). Extensive myocardial necrosis leads to the replacement of fibrosis. Regional ischemia is an aggravating mechanism for extensive fibrosis, as the abnormal intramural coronary artery with a thickened wall and narrowed lumen has been demonstrated histopathologically (2). Hence, myocardial fibrosis as an arrhythmogenic substrate causes non-smooth conduction, presenting as one or more notches on the QRS complex on ECG (26–28).

In addition, increased myocardial fibrosis not only results in conduction abnormalities, but also LV systolic dysfunction and heart failure (29, 30). Hence, it is not surprising that f-QRS showed a negative relationship with LVEF% in our DD group.

### Segmental Distribution of f-QRS and LGE in DD

Danon disease cardiomyopathy characteristically exhibits extreme myocardial thickening and significant fibrosis, predominantly in the LV free wall (8, 9). Although the exact mechanism of LV free wall scarring remains unclear, this distribution pattern of LGE in DD helps differentiate between sarcomeric HCM and other cardiomyopathies on CMR imaging (11, 31). In a study of patients with HCM (*n* = 60) (14), the presence of deep-notched QRS was associated with the location of LGE, predominantly in the septal wall (*n* = 18). In contrast, our DD cohort showed that the f-QRS score and LGE% were both distributed the least in the septum.

However, the segmental distribution of the f-QRS score and LGE, shown in Figure 3, seems not to be correlated well. The f-QRS score was highest in the inferior segments, and LGE% was predominant in the lateral segments. It is unclear whether f-QRS can be used to detect myocardial fibrosis in the corresponding LV segment. Studies on coronary artery disease (17) and HCM populations (14, 23) have shown that the location of f-QRS correlates with the site of scar tissue in the LV myocardium. Konno et al. (20) found that the distribution of f-QRS and LGE did not correlate well in their HCM cohort, in which f-QRS was also observed predominantly in the inferior leads, whereas LGE was present predominantly in the anterior and lateral segments.

Two possible reasons may explain this phenomenon. First, some of the f-QRS complexes in the inferior leads are benign ECG changes and may not represent myocardial abnormalities. In a study of 8,277 subjects, with no clinical or ECG evidence of cardiac disease (32), f-QRS was found most commonly in inferior leads (15.7% of the subjects), followed by anterior (2.9%) and lateral leads (0.8%). Moreover, even among subjects with a known cardiac disease, inferior f-QRS was not associated with an increased risk of mortality. Second, the inferior leads (II, III, and aVF) correspond to the involvement not only of the inferior wall but also the inferior part of the lateral wall, with or without the inferior part of the septum. Cino et al. (33) conducted a study on how ECG patterns correspond to different myocardial infarction locations detected by CMR. They found that the ECG abnormalities observed in the inferior leads correspond to involvement not only of the inferior wall but also the inferior part of the lateral wall, with or without the inferior part of the septum. Therefore, the fibrosis located in the inferior part of the lateral wall or the septum increased the presence of inferior f-QRS. These two explanations remind us that inferior f-QRS might over-evaluate the abnormalities in the inferior segments.

## Limitations

The primary limitation in our study was the small sample size; thus, the results should be validated in a larger DD population, although this disease is uncommon. Second, no proper control group was set for comparison to our patients with DD. To support the study results, an analysis from DD literature cohorts was also presented. Studies on correlations between f-QRS and myocardial fibrosis in the HCM cohort have also been discussed. Third, the present DD group was extremely unique: all subjects presented LGE on CMR and f-QRS on ECG. It might be necessary to validate the correlation between f-QRS and LGE negative subjects.

## Conclusion

The high prevalence of f-QRS in DD subjects raised our interest in investigating its importance to the cardiac status of this unique disease. In these subjects, f-QRS was significantly correlated with the extent of myocardial fibrosis and LV systolic dysfunction assessed by CMR imaging. The local distribution of leads with f-QRS seems more apparent in the LV free wall segments. These findings suggest that f-QRS might be a simple and cost-effective tool for screening the myocardial fibrosis burden and heart function in patients with DD.

## Data Availability Statement

The original contributions presented in the study are included in the article/Supplementary Material, further inquiries can be directed to the corresponding author/s.

## Ethics Statement

The studies involving human participants were reviewed and approved by the Ethics Committee of Guangdong Provincial People's Hospital. Written informed consent from the participants' legal guardian/next of kin was not required to participate in this study in accordance with the national legislation and the institutional requirements.

## Author Contributions

JX and YL: analysis and interpretation of data and drafting of the manuscript. XW, GL, ZT, and TL: image post-processing. WY and ZM: revising manuscript critically. HL and YC: conception and design and revising manuscript critically. YW, LZ, ML, and XL: investigation. All authors contributed to the article and approved the submitted version.

## Funding

This work was supported by the National Natural Science Foundation of China (Grant 81974262 and 81970288), Natural Science Foundation of Guangdong Province (Grant 2020A1515010650), and Guangdong Cardiovascular Institute Project (Grant 2020XXG009).

## Conflict of Interest

The authors declare that the research was conducted in the absence of any commercial or financial relationships that could be construed as a potential conflict of interest.

## Publisher's Note

All claims expressed in this article are solely those of the authors and do not necessarily represent those of their affiliated organizations, or those of the publisher, the editors and the reviewers. Any product that may be evaluated in this article, or claim that may be made by its manufacturer, is not guaranteed or endorsed by the publisher.

## Supplementary Material

Supplementary Table 1 Summary of CMR acquisition protocol details from different centers.

Supplementary Table 2 Summary of the ECG and of cardiac MRI characteristics of Danon disease in our cohort.

Supplementary Table 3 Summary of the ECG and of cardiac MRI characteristics of Danon disease in literature review.

Supplementary Table 4 Summary of mortality incidence and invasive treatment records in Danon disease of our cohort and the literature cohort.

Supplementary Figure 1 Comparison f-QRS characteristics in patient with Danon disease between our DD cohort, and the literature cohort. **(A)** Three patterns of f-QRS complexes were assessed on 12-lead ECG in this study (*n* = 15) and the kinds of literature (*n* = 36). The number on the bar presents the number of leads with f-QRS (proportion). **(B)** Segmental distribution of f-QRS complexes. The number on the bar presents the averaged f-QRS score of septal leads (V1–V2), anterior leads (V3–V4), lateral leads (I, aVL, V5, and V6), and inferior leads (II, III, and aVF).
