# Supplementary material for: Relationship Between Fragmented QRS Complex and Left Ventricular Fibrosis and Function in Patients With Danon Disease
Source: Front Cardiovasc Med. 2022 Feb 21;9:790917. doi: 10.3389/fcvm.2022.790917 (PMC8923125; doi:10.3389/fcvm.2022.790917)
Supplement: Supplementary Table 1 — Summary of CMR acquisition protocol details from different centers. [file Table_1.DOCX]

| **Supplementary Table 1: Summary of CMR acquisition protocol details from different centers.**  Note: CMR data of Danon cardiomyopathy were collected and analyzed from six tertiary centers in China. All CMR images were performed with 3.0 T and or 1.5T scanner using standard protocols. The detailed parameters by scanners were showed as following.   \| Centers and Patients \| Scanner (Field strength, T) \| Cine \| T2WI \| T2 +Fat-sat \| Perfusion \| LGE \| \| --- \| --- \| --- \| --- \| --- \| --- \| --- \| \| HUAXI^a^  (n=1) \| Simenzi trio (3.0 T) \| 30 phases;  Slice thickness: 8 mm  FOV: 240 mm;  matrix: 192*162;  TR/TE: 42.04/1.25  NEX 1;  50° flip angle; \| Slice thickness: 8 mm  FOV: 300mm;  matrix: 256*208;  TR: 761 ms;  TE: 30 ms;  echo train: 11;  NEX: 1;  180° flip angle; \| Slice thickness: 8 mm  FOV: 260 mm;  matrix: 256*208;  TR: 722 ms;  TE: 30 ms;  Echo train: 11;  TI: 220 ms  NEX: 1;  180° flip angle; \| Slice thickness:8 mm  FOV: 300 mm;  matrix: 176*132;  TR: 153.8ms;  TE: 1.06 ms;  Echo train: 1;  NEX: 1;  10° flip angle; \| Slice thickness: 8 mm  FOV: 250 mm;  matrix: 256*216  TR/TE: 660/3.68 ms;  Echo train: 1;  NEX: 1;  TI:380 ms;  20° flip angle; \| \| HUAXI^a^  (n=1) \| Simenzi skyra(3.0 T) \| 30 phases;  Slice thickness: 8 mm  FOV: 240 mm;  matrix: 192*162;  TR/TE: 41.38/1.28  NEX 1;  42° flip angle; \| Slice thickness: 8 mm  FOV: 300mm;  matrix: 256*208;  TR: 761 ms;  TE: 30 ms;  echo train: 11;  NEX: 1;  180° flip angle; \| Slice thickness: 8 mm  FOV: 260 mm;  matrix: 256*208;  TR: 722 ms;  TE: 30 ms;  Echo train: 11;  TI: 220 ms  NEX: 1;  180° flip angle; \| Slice thickness:8 mm  FOV: 300 mm;  matrix: 144*192;  TR: 147.78ms;  TE: 0.99 ms;  Echo train: 1;  NEX: 1;  10° flip angle; \| Slice thickness: 8 mm  FOV: 250 mm;  matrix: 144*192;  TR/TE: 807.20/1.09 ms;  Echo train: 1;  NEX: 1;  TI:320 ms;  40° flip angle; \| \| GPH^b^  （n=1） \| Philips Achieva (1.5 T) \| 30 phases;  Slice thickness: 8 mm  FOV: 40 cm;  matrix: 19*224;  60° flip angle;  TR/TE: 3.64 / 1.82  NEX 1;  50° flip angle; \| Slice thickness: 8 mm  FOV: 240mm;  matrix: 384*384;  TR: 2 R-R;  TE: 34.42 ms;  echo train: 50:  NEX: 1;  90° flip angle; \| Slice thickness: 8 mm  FOV: 240mm;  matrix: 352*352;  TR: 2 R–R;  TE: 70 ms;  Echo train: 28;  TI: 180 ms  NEX: 1;  90° flip angle; \| Slice thickness: 10 mm  FOV: 240mm;  matrix: 192*192;  TR: 3.25 ms;  TE: 1.22 ms;  Echo train: 29;  NEX: 1;  25° flip angle; \| Slice thickness: 8 mm  FOV: 380 to 420 mm;  matrix: 224*224  TR/TE: 3.76/1.88 ms;  Echo train: 65;  NEX: 2;  TI:280 ms;  50° flip angle; \| \| GPH^b^  （n=4） \| Philips Ingenia (3.0 T) \| 30 phases;  Slice thickness: 8 mm  FOV: 240 mm;  matrix: 256*256;  TR/TE: 3.44/1.72  NEX 1;  45° flip angle; \| Slice thickness: 8 mm  FOV: 220 mm;  matrix: 288*288;  TR: 2 R-R;  TE: 75 ms;  echo train: 25:  NEX: 1;  90° flip angle; \| Slice thickness: 8 mm  FOV: 220 mm;  matrix: 288*288;  TR: 2 R–R;  TE: 75 ms;  Echo train: 25;  TI: 220 ms  NEX: 1;  90° flip angle; \| Slice thickness: 10 mm  FOV: 300 mm;  matrix: 224*224;  TR: 2.51ms;  TE: 1.14 ms;  Echo train: 50;  NEX: 1;  20° flip angle; \| Slice thickness: 8 mm  FOV: 280 mm;  matrix: 256*256  TR/TE: 6.12/2.99 ms;  Echo train: 20;  NEX: 1;  TI:300 ms;  25° flip angle; \| \| FUWAI^c^  (n=2) \| Simenzi Avanto (1.5 T) \| 30 phases;  Slice thickness: 10 mm  FOV: 240 mm;  matrix: 216*256;  TR/TE: 41.04/1.51  NEX 1;  50° flip angle; \| Slice thickness: 5 mm  FOV: 300mm;  matrix: 256*208;  TR: 761 ms;  TE: 30 ms;  echo train: 11;  NEX: 1;  180° flip angle; \| Slice thickness: 5 mm  FOV: 260 mm;  matrix: 256*208;  TR: 722 ms;  TE: 30 ms;  Echo train: 11;  TI: 180 ms  NEX: 1;  180° flip angle; \| Slice thickness: 10 mm  FOV: 300 mm;  matrix: 144*126;  TR: 147.8ms;  TE: 1.08 ms;  Echo train: 1;  NEX: 1;  20° flip angle; \| Slice thickness: 8 mm  FOV: 250 mm;  matrix: 168*192  TR/TE: 717/1.04 ms;  Echo train: 1;  NEX: 1;  TI:300 ms;  20° flip angle; \| \| PUM^d^  (n=2) \| GE Signa EXCITE (3.0 T) \| 30 phases;  Slice thickness: 10 mm  FOV: 360 mm;  matrix: 512*512  TR/TE: 3.47/1.51  NEX 1;  45° flip angle; \| Slice thickness: 10 mm  FOV: 360mm;  matrix: 512*512  TR: 1000 ms;  TE: 4.58 ms;  echo train: 12;  NEX: 1;  90° flip angle; \| Slice thickness: 10 mm  FOV: 360mm;  matrix: 512*512  TR: 2 R–R;  TE: 72 ms;  Echo train: 25;  TI: 220 ms  NEX: 1;  180° flip angle; \| Slice thickness: 10 mm  FOV: 360 mm;  matrix: 256*256;  TR: 6.41ms;  TE: 1.46 ms;  Echo train: 4;  NEX: 1;  25° flip angle; \| Slice thickness: 10 mm  FOV: 260 mm;  matrix: 512*512  TR/TE: 6.62/3.10 ms;  Echo train: 1;  NEX: 1;  TI:200 ms;  20° flip angle; \| \| ANZHEN^e^  (n=2) \| Simenzi verio (3.0 T) \| 30 phases;  Slice thickness: 10 mm  FOV: 240 mm;  matrix: 216*256;  TR/TE: 41.04/1.51  NEX 1;  50° flip angle; \| Slice thickness: 8 mm  FOV: 300mm;  matrix: 256*208;  TR: 2 R-R;  TE: 70 ms;  echo train: 17;  NEX: 1;  180° flip angle; \| Slice thickness: 8 mm  FOV: 260 mm;  matrix: 256*208;  TR: 2 R–R;  TE: 70 ms;  Echo train: 25;  TI: 220 ms  NEX: 1;  180° flip angle; \| Slice thickness: 6 mm  FOV: 300 mm;  matrix: 144*192;  TR: 164ms;  TE: 1.01 ms;  Echo train: 50;  NEX: 1;  10° flip angle; \| Slice thickness: 8 mm  FOV: 250 mm;  matrix: 208*256  TR/TE: 433/1.53 ms;  Echo train: 1;  NEX: 1;  TI:340 ms;  25° flip angle; \| \| ANZHEN^e^  (n=1) \| Philips Ingenia (3.0 T) \| 30 phases;  Slice thickness: 10 mm  FOV: 270 mm;  matrix: 288*288;  TR/TE: 2.84/1.42  NEX 1;  45° flip angle; \| Slice thickness: 8 mm  FOV: 350mm;  matrix: 448*448;  TR: 1200 ms;  TE: 9 ms;  echo train: 16;  NEX: 1;  90° flip angle; \| Slice thickness: 8 mm  FOV: 300mm;  matrix: 336*336;  TR: 2 R–R;  TE: 75 ms;  Echo train: 25;  TI: 220 ms  NEX: 1;  90° flip angle; \| Slice thickness: 6 mm  FOV: 300 mm;  matrix: 224*224;  TR: 2.71ms;  TE: 1.23 ms;  Echo train: 51;  NEX: 1;  15° flip angle; \| Slice thickness: 10 mm  FOV: 300 mm;  matrix: 336*336  TR/TE: 6.11/2.99 ms;  Echo train: 19;  NEX: 1;  TI:320 ms;  25° flip angle; \| \| Anhui ^f^  (n=1) \| Philips Ingenia CX (3.0 T) \| 30 phases;  Slice thickness: 8 mm  FOV: 270 mm;  matrix: 288*288;  TR/TE: 3.09/1.54  NEX 1;  45° flip angle; \| Slice thickness: 8 mm  FOV: 300mm;  matrix: 288*288;  TR: 1052 ms;  TE: 30 ms;  echo train: 63;  NEX: 1;  90° flip angle; \| Slice thickness: 8 mm  FOV: 380mm;  matrix: 448*448;  TR: 2105;  TE: 75 ms;  Echo train: 20;  TI: 220 ms  NEX: 1;  90° flip angle; \| Slice thickness: 10 mm  FOV: 300 mm;  matrix: 224*224;  TR: 2.72ms;  TE: 1.24 ms;  Echo train: 51;  NEX: 1;  15° flip angle; \| Slice thickness: 9 mm  FOV: 280 mm;  matrix: 320*320  TR/TE: 6.12/3.00 ms;  Echo train: 21;  NEX: 1;  TI:320 ms;  25° flip angle; \|   ^a^ West China Hospital, Sichuan University, Chengdu, China.  ^b^ Guangdong Provincial People’s Hospital, Guangdong Academy of Medical Sciences, Guangzhou, China.  ^c^ Fuwai Hospital and National Center for Cardiovascular Diseases, Chinese Academy of Medical Sciences and Peking Union Medical College, Beijing, China.  ^d^ Peking Union Medical College Hospital, Chinese Academy of Medical Sciences, Beijing, China.  ^e^ Beijing Anzhen Hospital, No. 2 Anzhen Road, Chaoyang District, Beijing, China.  f Department of Radiology, the First Affiliated Hospital of Anhui Medical University, Hefei, Anhui Province, China. |
| --- | --- | --- | --- | --- | --- | --- | --- | --- | --- | --- | --- | --- | --- | --- | --- | --- | --- | --- | --- | --- | --- | --- | --- | --- | --- | --- | --- | --- | --- | --- | --- | --- | --- | --- | --- | --- | --- | --- | --- | --- | --- | --- | --- | --- | --- | --- | --- | --- | --- | --- | --- | --- | --- | --- | --- | --- | --- | --- | --- | --- | --- | --- | --- | --- | --- | --- | --- | --- | --- | --- |
