# Supplementary material for: Relationship Between Fragmented QRS Complex and Left Ventricular Fibrosis and Function in Patients With Danon Disease
Source: Front Cardiovasc Med. 2022 Feb 21;9:790917. doi: 10.3389/fcvm.2022.790917 (PMC8923125; doi:10.3389/fcvm.2022.790917)
Supplement: Supplementary Table 2 — Summary of the ECG and of cardiac MRI characteristics of Danon disease in our cohort. [file Table_2.DOCX]

| **Supplementary Table 2 Summary of the ECG and of cardiac MRI characteristics of Danon disease in our cohort** | | | | | | | | | | | | | | |
| --- | --- | --- | --- | --- | --- | --- | --- | --- | --- | --- | --- | --- | --- | --- |
| Case | Gender | Age at ECG, years | Symptoms | Gene mutation site | ECG characteristics | | | | | LV Phenotype and LGE location on CMR | | | | |
|  |  |  |  |  | Preexcitation | Other arrhythmia | LV high voltage | Fragmented QRS on leads | Fragmented QRS score | LV phenotype | LV wall thickness, mm | LGE main location | Treatment | Outcome |
| patient 1# | M | 19 | 1, 2, 3 | c.257_258delCC | - | CRBBB, AF, | + | 12 | 33 | HCM*† | 33 | LV, RV | Medicine | HF, Death |
| patient 2# | M | 19 | 1, 2, 3 | c.719_721dupAGC | + | NA | + | 12 | 22 | HCM*† | 24.3 | LV, RV | Medicine | HF |
| patient 3# | M | 17 | 2, 3 | c.29_35dupCGGGCTC | + | VCB | + | 11 | 15 | HCM*† | 28.1 | LVFW | Medicine | HF, Death |
| patient 4# | M | 15 | 1 | c.1057C>T | + | NA | + | 12 | 16 | HCM*† | 32.5 | LV, RV | Medicine | HF, Death |
| patient 5# | M | 24 | 2 | c.35C>A | + | NA | - | 12 | 15 | DCM | 11.1 | LV, RV | Medicine | HF |
| patient 6# | M | 23 | 1 | c.973delG | + | NA | - | 9 | 11 | HCM† | 14.5 | LVFW | RFA | HF |
| patient 7# | M | 19 | 1, 3 | c.973dupC | - | CLBBB | + | 8 | 9 | HCM*† | 20.9 | LVFW | Medicine | HF, Death |
| patient 8# | M | 21 | 2 | c.973delC | + | AF | + | 7 | 9 | HCM*† | 13 | LVFW | Medicine | HF |
| patient 9# | F | 44 | 2 | c.64+1G>A | + | AF | - | 7 | 8 | HCM*† | 14 | LVFW | Medicine | HF |
| patient 10 | M | 16 | 2 | c.963G>A | + | NA | + | 6 | 6 | HCM* | 22 | LVFW | Medicine | Alive |
| patient 11#§ | M | 15 | 1 | c.257_258delCC | + | NA | + | 4 | 4 | HCM*† | 21.1 | LVFW | Medicine | HF, Death |
| patient 12# | M | 23 | 2 | c.65-2A>G | + | SA | + | 3 | 3 | HCM* | 30.8 | LVFW | RFA | Alive |
| patient 13#§ | M | 14 | 3 | c.808_809 insG | + | NA | + | 3 | 3 | HCM* | 15.5 | LVFW | Medicine | HF, Death |
| patient 14# | M | 20 | 3 | c.64+1G>A | + | NA | - | 2 | 2 | HCM*† | 29.9 | LVFW | ICD | HF |
| patient 15# | M | 17 | 1, 2 | c.877C>T | + | APB | + | 2 | 2 | HCM*† | 15.9 | LVFW | RFA | HF |

Note. + indicates the presence of the feature; - indicates the absence of the feature. Male, M; Female, F; Electrocardiography, ECG; Ventricular conduction block, VCD; Sinus arrhythmia, SA; Atrial fibrillation, AF; Atrial premature beats, APB; Complete left bundle branch block, CLBBB; Hypertrophic cardiomyopathy, HCM; Dilated cardiomyopathy, DCM; Left ventricle, LV; LV free wall, LVFW; Right ventricle, RV; Heart failure, HF; Radiofrequency ablation, RFA; Implantable cardioverter defibrillator, ICD; Non-available, NA;1, chest pain; 2, chest tightness and short of breath; 3, syncope;

*Presence of advanced HCM phenotype that manifested as LV dilatation at the time of cardiac MRI examination;

†Presence of advanced HCM phenotype that manifested with systolic LV dysfunction at the time of cardiac MRI examination;

# These 14 cases were previously reported CMR results by our teammate (Wei et al, doi:10.1148/radiol.2021203996). Their published MRI images are not shown in this article.

§The two patients in the study by Liu et al (doi:10.1093/eurheartj/ehr438) were the same patients as in our study. Their ECGs were not displayed in this article.
