# Supplementary material for: Relationship Between Fragmented QRS Complex and Left Ventricular Fibrosis and Function in Patients With Danon Disease
Source: Front Cardiovasc Med. 2022 Feb 21;9:790917. doi: 10.3389/fcvm.2022.790917 (PMC8923125; doi:10.3389/fcvm.2022.790917)
Supplement: Supplementary Table 3 — Summary of the ECG and of cardiac MRI characteristics of Danon disease in literature review. [file Table_3.DOCX]

| **Supplementary Table 3 Summary of the ECG and of cardiac MRI characteristics of Danon disease in literature review** | | | | | | | | | | | | |
| --- | --- | --- | --- | --- | --- | --- | --- | --- | --- | --- | --- | --- |
| Case (reference) | Gender | Age at ECG, years | Symptoms | ECG characteristics | | | | | LV Phenotype and LGE location on CMR | | | |
|  |  |  |  | Preexcitation | Other arrhythmia | LV high voltage | Fragmented QRS on leads | Fragmented QRS score | LV phenotype | LV wall thickness, mm | LGE main location | Treatments and Outcome |
| 1 (1) | M | 17 | Chest discomfort | - | NA | + | 12 | 12 | HCM | 25 | NA | ICD |
| 2 (2) | M | NA | NA | - | NA | + | 0 | 0 | NA | NA | NA | NA |
| 3 (3) | M | 14 | NA | + | NA | + | 7 | 7 | HCM | 43 | NA | NA |
| 4 (3) | M | 23 | NA | - | NA | - | 0 | 2 | Normal | Normal | NA | NA |
| 5 (4) | M | 15 | NA | - | NA | + | 5 | 6 | HCM | 39 | NA | NA |
| 6 (5) | M | 14 | Syncope | + | NA | + | 5 | 5 | HCM | 60 | NA | Died at 23y |
| 7 (6) | M | 17 | Palpitation | - | AV block | + | 6 | 8 | HCM | 30 | NA | ICD |
| 8 (7) | M | 12 | Fatigue | + | AV block | + | 1 | 1 | HCM | 27 | NA | NA |
| 9 (8) | M | 8 | Palpitation | - | LBBB | + | 4 | 4 | HCM | 26 | NA | RFA, ICD, Died at 14y |
| 10 (9) | F | 13 | NA | + | NA | + | 1 | 1 | Normal | 7 | NA | NA |
| 11 (10) | F | 15 | Palpitation | + | NA | + | 0 | 0 | HCM | 24 | LVFW | ICD |
| 12 (11) | M | 5 | Fatigue | + | NA | + | 0 | 0 | HCM | 17 | NA | NA |
| 13 (12) | M | 14 | Fatigue | + | NA | + | 7 | 7 | HCM | 26 | LVFW | NA |
| 14 (13) | M | 11 | Palpitation | + | LBBB | + | 10 | 12 | HCM | NA | NA | Died at 15y |
| 15 (14) | F | - | NA | - | AF | - | 8 | 8 | DCM | NA | NA | NA |
| 16 (14) | M | 18 | Chest tightness | + | APB, CLBBB | + | 6 | 7 | HCM | 15 | LVFW | NA |
| 17 (14) | M | 13 | NA | + | NA | - | 2 | 3 | NA | NA | NA | NA |
| 18 (15) | M | 28 | Fatigue | + | NA | + | 1 | 0 | HCM | 21 | NA | NA |
| 19 (16) | M | 21 | Fatigue | + | NA | + | 11 | 15 | HCM | 27 | LVFW | Died at 21y |
| 20 (17) | M | 13 | Short of breath | + | NA | + | 8 | 15 | HCM | 19.4 | NA | Died at 13y |
| 21 (18) | M | 12 | Syncope | + | NA | + | 0 | 0 | HCM | 14 | NA | NA |
| 22 (18) | M | 12 | SMW | + | VT | + | 10 | 14 | HCM | 14 | LVFW | RFA, Died at 21y |
| 23 (19) | F | 11 | HF symptom | + | NA | + | 2 | 2 | HCM | 41 | NA | Died at 35y |
| 24 (19) | M | 28 | Chest pain | + | NA | + | 11 | 11 | HCM | 22 | NA | ICD |
| 25 (20) | M | 17 | HF | + | NA | + | 12 | 17 | HCM* | 22 | NA | NA |
| 26 (21) | F | 23 | II | + | NA | + | 6 | 10 | HCM* | 32 | NA | HT at 23y, Died at 28y |
| 27 (22) | M | 19 | Exertional dyspnea | + | AVB | + | 6 | 11 | HCM | 19 | NA | NA |
| 28 (22) | M | 23 | Chest pain | + | NA | + | 3 | 3 | HCM | 33 | NA | NA |
| 29 (22) | M | 17 | Cardiac arrest | + | VF | + | 9 | 10 | HCM | 29 | NA | NA |
| 30 (23) | F | 25 | Palpitation | + | AF | + | 2 | 2 | HCM | NA | NA | Died at 52y |
| 31(24) | F | 20 | Acute HF | + | NA | + | 12 | 16 | HCM* | NA | NA | HT at 20y |
| 32 (24) | F | 25 | NA | + | NA | + | 4 | 4 | DCM | NA | NA | ICD, HT at 25y |
| 33 (24) | F | 20 | Syncope | + | NA | + | 7 | 7 | HCM | 18 | NA | ICD at 21Y |
| 34 (25) | M | 16 | HF symptom | + | NA | + | 8 | 9 | HCM* | 18 | NA | ICD at 16 y, Died at 17y |
| 35 (26) | M | 21 | Exertional dyspnea | + | CLBBB | + | 6 | 6 | HCM* | 13 | LV+RV | ICD |
| 36 (27) | M | 18 | SMW, II | - | NA | + | 10 | 15 | HCM | 35 | NA | ICD |
| **Total**  **(N=36)** | 27:9† | 17(5,28) ‡ | - | 28/36 | 12/36 | 33/36 | 6(0, 12) ‡ | 7(0, 17) ‡ | HCM 30/36 | 24.5(7, 60) ‡ | CMR and LGE+:6 | RFA:2  ICD:10  HT:3  Death: 10 |
| **Our study**  **(N=15)** | 14:1† | 19(14,44) ‡ | - | 13/15 | 6/15 | 11/15 | 7(2, 12) ‡ | 9(2, 33) ‡ | HCM 14/15 | 21 (11, 33) ‡ | CMR and LGE+:15 | RFA:3  ICD:1  HT:0  Death: 6 |

Note. + indicates the feature's presence; - indicates the feature's absence. Male, M; Female, F; Electrocardiography, ECG; Skeletal muscle weakness, SMW; Intellectual impairment, II; Heart failure, HF; Atrial premature beats APB; Ventricular tachycardia VT; Ventricular fibrillation, VF; Atrial fibrillation, AF; Atrioventricular block, AVB; Complete left bundle branch block, CLBBB; Left ventricle, LV; LV free wall, LVFW; Right ventricle, RV; Hypertrophic cardiomyopathy, HCM; Dilated cardiomyopathy, DCM; Radiofrequency ablation, RFA; Implantable cardioverter defibrillator implantation, ICD; Heart transplantation, HT; Non-available, NA;

*Presence of advanced HCM phenotype that manifested with LV dilatation at the time of cardiac MRI;

†Data presented as number of male / number of female patients;

‡ Data presented as median (range);

**References**

1. Zaki A, Zaidi A, Newman WG, et al. Advantages of a subcutaneous implantable cardioverter-defibrillator in LAMP2 hypertrophic cardiomyopathy. J Cardiovasc Electrophysiol. 2013;24(9):1051-3.

2. Konrad T, Sonnenschein S, Schmidt FP, et al. Cardiac arrhythmias in patients with Danon disease. Europace. 2017;19(7):1204-10.

3. A ́ ngela Lo ́ pez-Sainz, Joel Salazar-Mendiguchı ́a, Ana Garcı ́a-A ́ lvarez, et al. Clinical Findings and Prognosis of Danon Disease. An Analysis of the Spanish Multicenter Danon Regist ry. Rev Esp Cardiol (Engl Ed). 2018.

4. Mueller P, Attenhofer Jost CH, Rohrbach M, et al. Cardiac disease in children and young adults with various lysosomal storage diseases: Comparison of e chocardiographic and ECG changes among clinical groups. Int J Cardiol Heart Vessels. 2013;2:1-7.

5. Maron BJ, Roberts WC, Arad M, et al. Clinical outcome and phenotypic expression in LAMP2 cardiomyopathy. Jama. 2009;301(12):1253-9.

6. Novelli V, Bisignani A, Pelargonio G, et al. Clinical utility of genetic testing in the early diagnosis of Danon disease mimicking hypertrophic cardiomyopathy: a case report. BMC Cardiovasc Disord. 2020;20(1):156.

7. Lines MA, Hewson S, Halliday W, et al. Danon Disease Due to a Novel LAMP2 Microduplication. JIMD Rep. 2014;14:11-6.

8. D'Souza RS, Mestroni L, Taylor MRG. Danon disease for the cardiologist: case report and review of the literature. J Community Hosp Intern Med Perspect. 2017;7(2):107-14.

9. Taylor MR, Ku L, Slavov D, et al. Danon disease presenting with dilated cardiomyopathy and a complex phenotype. J Hum Genet. 2007;52(10):830-5.

10. Vago H, Somloi M, Toth A, et al. Danon disease: a rare cause of left ventricular hypertrophy with cardiac magnetic resonance follow-up. Eur Heart J. 2016;37(21):1703.

11. Cottinet SL, Bergemer-Fouquet AM, Toutain A, et al. Danon disease: intrafamilial phenotypic variability related to a novel LAMP-2 mutation. J Inherit Metab Dis. 2011;34(2):515-22.

12. Hashida Y, Wada T, Saito T, et al. Early diagnosis of Danon disease: Flow cytometric detection of lysosome-associated membrane protein-2 -negative leukocytes. J Cardiol. 2015;66(2):168-74.

13. Balmer C, Ballhausen D, Bosshard NU, et al. Familial X-linked cardiomyopathy (Danon disease): diagnostic confirmation by mutation analysis of the LAMP2gene. Eur J Pediatr. 2005;164(8):509-14.

14. Zhou N, Cui J, Zhao W, et al. A family with Danon disease caused by a splice site mutation in LAMP2 that generates a truncated protein. Mol Genet Genomic Med. 2019;7(3):e561.

15. Fanin M, Nascimbeni AC, Fulizio L, et al. Generalized lysosome-associated membrane protein-2 defect explains multisystem clinical involvement and allows leukocyte diagnostic screening in Danon disease. Am J Pathol. 2006;168(4):1309-20.

16. Le DD, Alvarez P, Barrios R, et al. Hypertrophic Cardiomyopathy with Unusual Extensive Scarring Pattern: Danon Disease. Methodist Debakey Cardiovasc J. 2016;12(4):227-9.

17. Fu L, Luo S, Cai S, et al. Identification of LAMP2 Mutations in Early-Onset Danon Disease With Hypertrophic Cardiomyopathy by Targeted Next-Generation Sequencing. Am J Cardiol. 2016;118(6):888-94.

18. Yang Z, Funke BH, Cripe LH, et al. LAMP2 microdeletions in patients with Danon disease. Circ Cardiovasc Genet. 2010;3(2):129-37.

19. Samad F, Jain R, Jan MF, et al. Malignant cardiac phenotypic expression of Danon disease (LAMP2 cardiomyopathy). Int J Cardiol. 2017;245:201-6.

20. Dougu N, Joho S, Shan L, et al. Novel LAMP-2 mutation in a family with Danon disease presenting with hypertrophic cardiomyopathy. Circ J. 2009;73(2):376-80.

21. Bottillo I, Giordano C, Cerbelli B, et al. A novel LAMP2 mutation associated with severe cardiac hypertrophy and microvascular remodeling in a female with Danon disease: a case report and literature review. Cardiovasc Pathol. 2016;25(5):423-31.

22. Liu Y, Chen X, Wang F, et al. Prevalence and clinical characteristics of Danon disease among patients with left ventricular hypertrophy and concomitant electrocardiographic preexcitation. Mol Genet Genomic Med. 2019;7(5):e638.

23. Miani D, Taylor M, Mestroni L, et al. Sudden death associated with danon disease in women. Am J Cardiol. 2012;109(3):406-11.

24. Mulder BA, Hoedemaekers YM, van den Berg MP, et al. Three female patients with Danon disease presenting with predominant cardiac phenotype: a case series. Eur Heart J Case Rep. 2019;3(3):ytz132.

25. Tanidir C, Tanidir IC, Tuzcu V. Treatment of depression in an adolescent with cardiomyopathy and arrhythmia. Cardiol Young. 2015;25(7):1418-20.

26. Tada H, Harimura Y, Yamasaki H, et al. Utility of real-time 3-dimensional echocardiography and magnetic resonance imaging for evaluation of Danon disease. Circulation. 2010;121(17):e390-2.

27. Zannad N, Pierre B, Cosnay P, et al. A young patient with Danon disease receives two ICD shocks: why? Pacing Clin Electrophysiol. 2010;33(5):618-9
