# Supplementary material for: Relationship Between Fragmented QRS Complex and Left Ventricular Fibrosis and Function in Patients With Danon Disease
Source: Front Cardiovasc Med. 2022 Feb 21;9:790917. doi: 10.3389/fcvm.2022.790917 (PMC8923125; doi:10.3389/fcvm.2022.790917)
Supplement: Supplementary Table 4 — Summary of mortality incidence and invasive treatment records in Danon disease of our cohort and the literature cohort. [file Table_4.DOCX]

**Supplementary Table 4 Summary of mortality incidence and invasive treatment records in Danon disease of our cohort and the literature cohort.**

|  | f-QRS score< 9 | f-QRS score≥ 9 | P value * |
| --- | --- | --- | --- |
| Death outcome |  |  |  |
| Our cohort (n=15) | 2/7, 29% | 4/8, 50% | 0.608 |
| Literature cohort (n=36) | 4/23, 17% | 6/13, 46% | 0.073 |
| Invasive treatment (ICD/RFA/HT) + |  |  |  |
| Our cohort (n=15) | 3/7, 43% | 1/8, 13% | 0.282 |
| Literature cohort (n=36) | 6/23, 26% | 7/13, 54% | 0.097 |

Note. * Fisher’s exact test was used to compare the incidence of adverse events between the two groups; + If the case has one or more invasive treatments in the brackets, it will count as one. Radiofrequency ablation, RFA; Implantable cardioverter defibrillator implantation, ICD; Heart transplantation, HT
