# Supplementary figures and images for: Relationship Between Fragmented QRS Complex and Left Ventricular Fibrosis and Function in Patients With Danon Disease
Source: Front Cardiovasc Med. 2022 Feb 21;9:790917. doi: 10.3389/fcvm.2022.790917 (PMC8923125; doi:10.3389/fcvm.2022.790917)

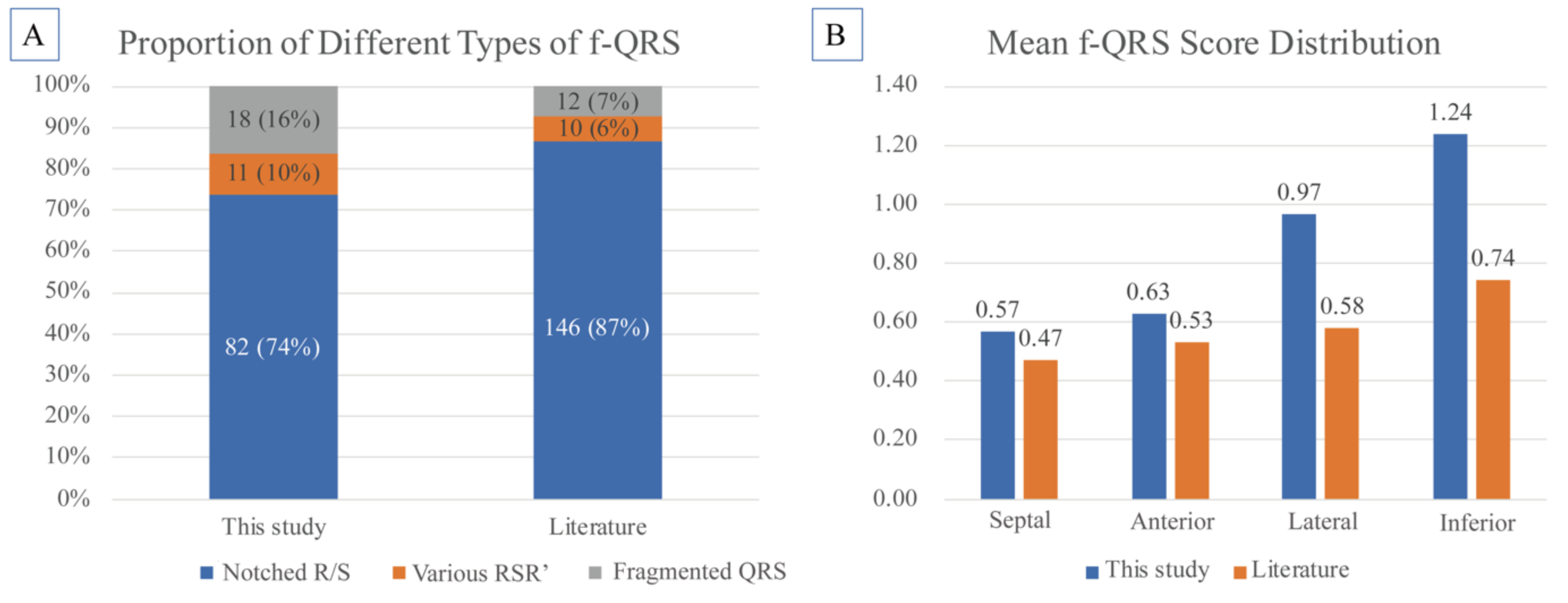

Supplement: Supplementary Figure 1 — Comparison f-QRS characteristics in patient with Danon disease between our DD cohort, and the literature cohort. (A) Three patterns of f-QRS complexes were assessed on 12-lead ECG in this study (n = 15) and the kinds of literature (n = 36). The number on the bar presents the number of leads with f-QRS (proportion). (B) Segmental distribution of f-QRS complexes. The number on the bar presents the averaged f-QRS score of septal leads (V1–V2), anterior leads (V3–V4), lateral leads (I, aVL, V5, and V6), and inferior leads (II, III, and aVF). [file Image_1.TIF]
